# An efficient breast cancer classification model using bilateral filtering and fuzzy convolutional neural network

**DOI:** 10.1038/s41598-024-56698-8

**Published:** 2024-03-15

**Authors:** A. Abdul Hayum, J. Jaya, R. Sivakumar, B. Paulchamy

**Affiliations:** 1 Electronics and Communication Engineering, Hindusthan Institute of Technology, Coimbatore, 641032 India; 2 Electronics and Communication Engineering, Hindusthan College of Engineering and Technology, Coimbatore, 641032 India; 3 School of Electronics Engineering, Vellore Institute of Technology, Vellore, India

**Keywords:** Computer-aided detection, Breast cancer, Modified fuzzy C means clustering, Mutation chicken swarm optimization, Fuzzy convolutional neural network, Cancer, Diseases, Health care, Engineering

## Abstract

BC (Breast cancer) is the second most common reason for women to die from cancer. Recent workintroduced a model for BC classifications where input breast images were pre-processed using median filters for reducing noises. Weighed KMC (K-Means clustering) is used to segment the ROI (Region of Interest) after the input image has been cleaned of noise. Block-based CDF (Centre Distance Function) and CDTM (Diagonal Texture Matrix)-based texture and shape descriptors are utilized for feature extraction. The collected features are reduced in counts using KPCA (Kernel Principal Component Analysis). The appropriate feature selection is computed using ICSO (Improved Cuckoo Search Optimization). The MRNN ((Modified Recurrent Neural Network)) values are then improved through optimization before being utilized to divide British Columbia into benign and malignant types. However, ICSO has many disadvantages, such as slow search speed and low convergence accuracy and training an MRNN is a completely tough task. To avoid those problems in this work preprocessing is done by bilateral filtering to remove the noise from the input image. Bilateral filter using linear Gaussian for smoothing. Contrast stretching is applied to improve the image quality. ROI segmentation is calculated based on MFCM (modified fuzzy C means) clustering. CDTM-based, CDF-based color histogram and shape description methods are applied for feature extraction. It summarizes two important pieces of information about an object such as the colors present in the image, and the relative proportion of each color in the given image. After the features are extracted, KPCA is used to reduce the size. Feature selection was performed using MCSO (Mutational Chicken Flock Optimization). Finally, BC detection and classification were performed using FCNN (Fuzzy Convolutional Neural Network) and its parameters were optimized using MCSO. The proposed model is evaluated for accuracy, recall, f-measure and accuracy. This work’s experimental results achieve high values of accuracy when compared to other existing models.

## Introduction

BC is a kind of cancer that develops in the breast. Cancer is brought on by the uncontrolled division or growth of cells. Typically, BC cells may be seen on radiographs and are the origin of tumors^1^. BC has emerged as leading mortality cases amongst women. Affecting 10% of women at some point in their life, BC is one of the most prevalent malignancies in women and is continuously linked to extraordinarily high morbidity and fatality rates. It is the second largest cause of mortality for women, after lung cancer. BC accounts for 25% of all malignancies and 12% of all new cases in women, respectively. Tumour grading can be used to identify BC^2^.

The two different types of tumours that are present in BC instances are malignant and benign. Cancerous tumours proliferate more quickly than benign ones. Clinicians want a trustworthy diagnostic method to distinguish between these tumours^3,4^. It will be possible to treat BC more effectively and boost survival rates if BC is found early with screening and diagnostic mammography. Unfortunately, identifying suspected irregularities might have a significant degree of inaccuracy due to the human aspect involved in the screening process. Because radiologists primarily rely on visual inspection, this mistake rate is increased^5,6^.

Radiologists might rapidly grow fatigued and lose vital signs when manually screening a large number of mammograms^7^. Massive attempts are being undertaken to automate the mammography screening process in order to offset these impacts. Research on automated BC mammography and CAD screening is extensive. In recent years, British Columbia has implemented a number of forecasting techniques. A variety of classification methods were utilised, including RF (Random Forest), SVM (Support Vector Machine), Adaboost Classifier, KNN (K Nearest Neighbour), and XGboost Classifier. To increase accuracy, a powerful model is still necessary.

Recent efforts have presented the BC classification model as a solution to the aforementioned issues where input breast image noises were in pre-processing using median filters. Subsequently, weighed KMC and ROI segmentation eliminated input image noises. Block-based CDF and CDTM-based texture and shape descriptors are employed for feature extraction. Dimensionality reduction of the retrieved features is carried out using KPCA. The calculation of the relevant feature selection makes use of ICSO. Following these operatrions, optimisationsfinetuned MRNN outcomes for categorizing BC into benign and malignant types. HoweverICSOhas many disadvantages, such as slow search speed and low convergence accuracy and training an MRNN is a completely tough task.

In this study, preprocessing is done via bilateral filtering to eliminate noise from the input image in order to prevent these issues. Contrasts are stretched for enhancing image qualities and assist in determining MFCM, ROI segmentations. For feature extractions, colour histograms and shape description algorithms based on CDTM and CDF are used. KPCA is used to minimise the size once the features have been extracted. Utilising Mutant Chicken Soup Optimisation (MCSO), features were chosen. Finally, FCNN was used to identify and classify BC, while MCSO was used to optimise the parameters of FCNN.

## Related works

GLCM (grayscale co-occurrence matrix) and SVM were used in tandem by Sarosa et al.^8^ to identify patients with benign malignancy from mammography images. This study used mammography data to determine the ideal GLCM angle for cases with BC categorization. This work used managed Mammogram subsets of Digital Database Screening Mammography (CBIS-DDSM) dataset and accuracy of 63.03% with specificity of 89.01% were obtained in experimental findings.

Zebari et al.^9^, to calculate ROI, provide a better threshold-based and trainable segmentation model. Based on thresholding methods and ML (Machine Learning), a combined segmentation method for the breast and pectoral muscle borders in mammography images has been devised. The breast area is emphasised by eliminating bands from the wavelet transform in order to estimate the breast borders. A novel thresholding method was employed to define the initial breast limit. By deleting tiny items, the morphological and masking techniques used to fix the border are overstated. The development of effective and precise ML algorithms for segmentation has advanced significantly in the realm of medical imaging. The crucial role of ML approaches in developing a more effective and accurate segmentation method is stressed in the literature. The pectoral muscle area and ROI were identified in this study using a machine learning approach based on the HOG (Oriented Gradient Graph) function and neural network classifiers. Using 322, 200, and 100 mammography images from the mini-MIAS, IN breast, and BCDR (Mammography Image Analysis Association) databases, the suggested segmentation approach was evaluated. Relevant BC digital storage). In order to compare test results with manual segmentation based on several texture properties. Additionally, the evaluation and comparison of the pectoralis muscle division and the chest area limitation were carried out independently. According to experimental findings, the chest and chest muscle delineation methods are 100% accurate (INbreast), 98.01% accurate (mini-MIAS), and 98.13% accurate (mini-MIAS). Respectively, 99.8% and 99.5% (BCDR). The breast segment limit accuracy for the proposed study was 99.31 percent on average, while the pectoral muscle segmentation accuracy was 98.64 percent on average. After enhancing the threshold methodology for basal segmentation and developing the ML technique for pectoral muscle segmentation, the suggested method's total ROI performance demonstrates enhanced accuracy. This article also presents fundamental information to assess general similarity. This study can be made available in the clinic as a helpful tool for determining BC.

Liu et colleagues^10^ proposed AlexNets for differentiating BC from other breast diseases. They pre-trained their model on the ImageNet dataset for enhancements. Their usage of cross entropy loss functions with punishments in output distributions to provide appropriate forecasts for distribution uniformity. Their repeated test on Break His, IDC, and UCSB datasets resulted in better outcomes than more sophisticated methods at various levels of magnifications, according to experimental findings. It is ideal for computer-aided clinical diagnosis systems in histology because to its stability and great generalisation.

First fully connected layer CNN (FCLF-CNN) was a suggested architecture by Liu et al.^7^ in their study where fully connected layers were merged before the first convolution layer and utilizing fully connected layers as approximators or encoders to transform unprocessed data into representations with greater locality. I trained four different FCLF-CNN models and combined them to produce a set of FCLF-CNN in order to improve performance. Then, using fivefold cross-validation, I applied it to the WDBC and WBCD datasets to acquire the result. The findings demonstrate that, for both datasets, FCLF-CNN outperforms pure multilayer perceptron and pure CNN in terms of classification performance. For WDBC, the FCLF-CNN kit obtained accuracy (99.28%), sensitivity (98.65), and specificity (99.57%) while for WBCD the corresponding values obtained for 98.71%, 97.60, and 99.43%, respectively. The results of both data sets are competitive with the results of other studies.

Hamed et al.^11^ suggested a CAD system based on YOLOV4 for detections of lesions from cropped mammograms and their subsequent classifications to identify pathological types. Their suggested approach's three steps were applied on digital mammograms of the INbreast dataset. The mammography is divided into thin, overlapping slices after being preprocessed to eliminate any artefacts. Second, volumes are positioned using the YOLO-V4 model setup to identify entire mammograms as well as clipped parts. Third, the local lesions extracted with YOLO, were classified using ResNet, VGG, Inception, etc. The impact of employing the YOLO-V4 detector with two detection lines of complete mammography and partial clipping in one trial to prevent data loss has been experimentally shown by the suggested technique. If there have been significant mammography size changes. Our methodology outperforms recently established BC detection techniques in terms of overall accuracy, identifying the bulk' location with a precision of roughly 98% and additionally, the capacity to accurately differentiate between benign and malignant tumours with 95% accuracy rate.

Zhang et al.^12^ suggested a classification technique for CNN-based synthesis and mammography. With permission from the institutional review board of the University of Kentucky, we acquired more than 3,000 mammography and image synthetic data. Different CNN models were built to categorise both 2D and 3D composite techniques of mammography, and each classifier was assessed using the real values produced by the biopsy findings. An experienced radiologist validated the histology and two-year negative mammography follow-up. Our findings showed that the CNN-based models we created and improved using transfer learning and data augmentation have a decent chance of detecting BC automatically. Based on data from optical synthesis and mammography.

Rastghalam and Pourghassem^13^ suggested texture feature extractions based on the MRF (Markov Random Fields), and LBP (Binary Patterns) in images. The study sought to extract breast textures in their MRF-based probable texture functions with suitable system definitions and neighbourhood groups in constructing new possible functions. The suggested schema’s tests on different thermal imaging datasets achieved false negative rates of 8.3% with 5% false positives.

## Proposed methodology

**Figure 1:**
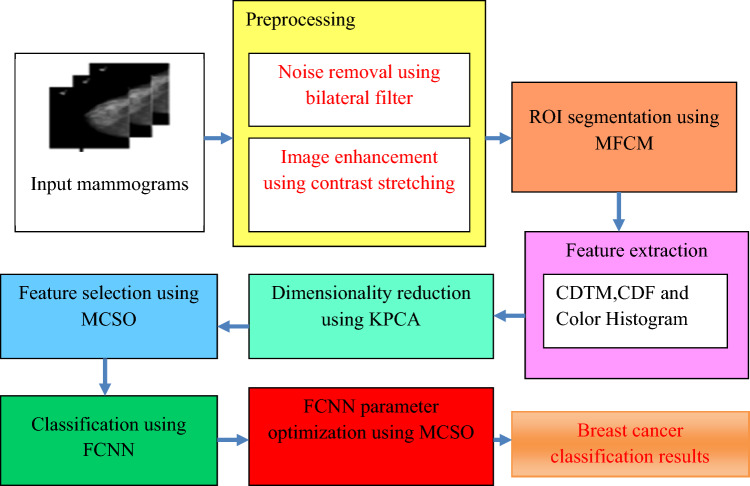
Overall architecture of the proposed model.

This section discusses the seven phases of the proposed model. First one is preprocessing in which bilateral filtering based noise removal and image enhancement using contrast stretching are done, Second one is region of interest extraction using modified FCM (fuzzy C means) clustering, third one is feature extraction based on Block-based CDTM, CDF based shape descriptor and Color Histogram, fourth one is Dimensionality reduction using Kernel Principal Component Analysis, fifth one is feature selection using MCSO, classification using FCNN and FCNN parameter optimization using MCSO.Overall architecture of this work’s model is depicted as Fig. 1.

### Noise removal using bilateral filter

Input images have more noise so need to remove this first. In this work bilateral filtering is used. An edge-preserving smoothing technique known as bilateral filtering is a non-repeating procedure. One of the mean filters used in this context is the median filter. Regardless of the existence of noise or edges, these convolutions can dramatically diminish edge information, blurring the image altogether. The nonlinear bilateral filter was developed to address the aforementioned issue. Bilateral filter using linear Gaussian $\mathcalligra{l}\mathcalligra{g}$ as the first step for smoothing:

1 $$\mathcalligra{l}\mathcalligra{g}\left(x\right)=\left(f\mathcal{*}\mathcal{L}{\mathcal{G}}_{s}\right)\left(x\right)$$

**Figure 2:**
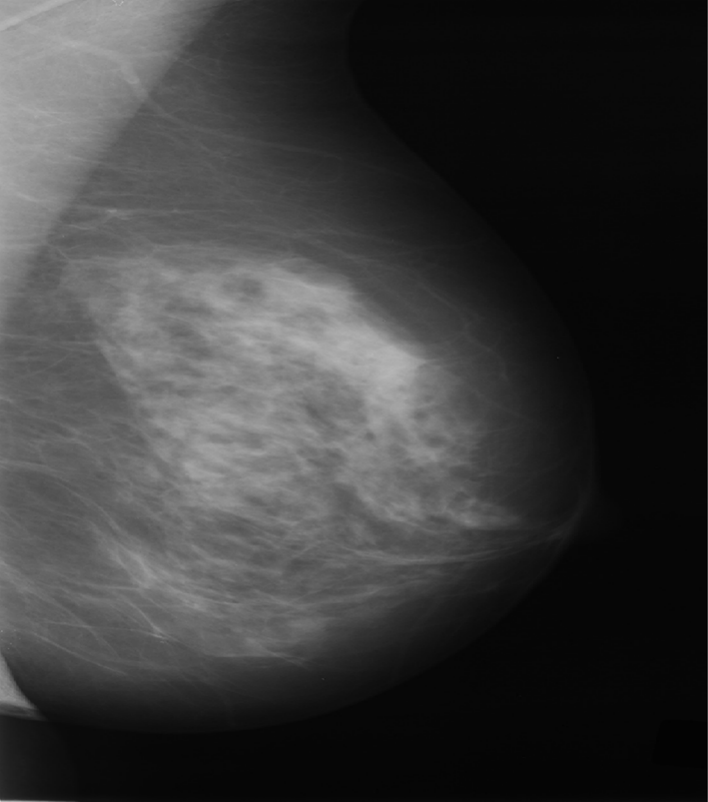
Pre-processing image for DDSM.

The weight for $f\left(y\right)$ equals $\mathcal{L}{\mathcal{G}}_{s}\left(x-y\right)$ and is only dependent on the spatial distance $\parallel x-y\parallel$. The bilateral filter adds a weighting term $wt$ that depends on the total distance $f\left(y\right)-f\left(x\right)$. Figure 2 shows the pre-processing image for DDSM dataset.

This results in:

2 $$\mathcalligra{l}\mathcalligra{g}\left(x\right)=\int f\left(y\right)\mathcal{*}\mathcal{L}{\mathcal{G}}_{s}\left(x-y\right)*wtdy$$

Be aware that as the weights directly depend on the image's values, normalisation is necessary in order to ensure that the total weights equal 1. The primary benefit of bilateral filtering is the removal of noise by image smoothing.

### Image enhancement using contrast stretching

The image should be better when the noise has been removed. Stretching the contrast is employed to improve in this piece. Contrast enhancement techniques are used to increase the range of brightness values in an image so that the image can be properly shown in the analyst's preferred way. Images' contrast levels might differ because too bad illumination or improper acquisition sensor settings. In order to make up for the challenges in image capture, the contrast of the image must be adjusted. Stretching the contrast is done to broaden the grayscale's dynamic range in the processed image. The goal is to alter the image's grayscale's dynamic range. The simplest contrast stretching algorithm, linear contrast tension, increases the dynamic range throughout the image spectrum to stretch the pixel values of low contrast or high contrast images. Between 0 and (L − 1).

### RoI segmentation using modified FCM clustering

**Figure 3:**
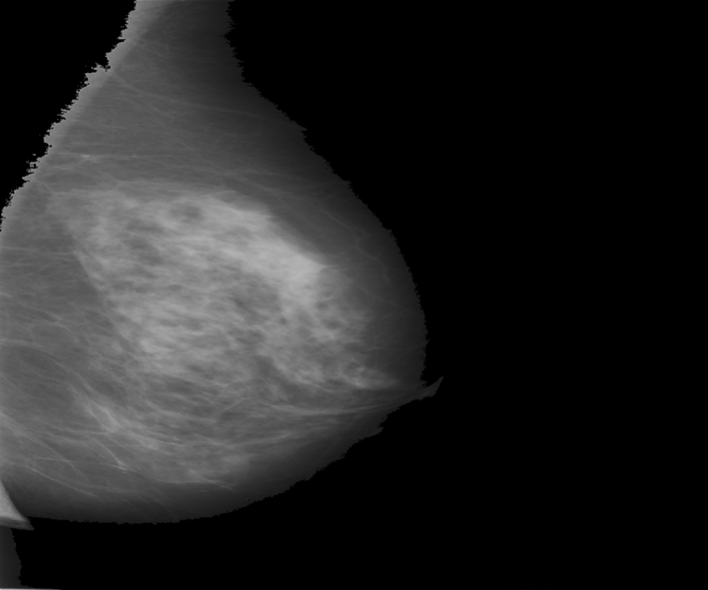
Segmentation for DDSM dataset.

These preprocessed images are forwarded for ROI segmentation when preprocessing is finished. The ROI is segmented in this study via clustering due to the changed c opacity. FCM (Fuzzy c-means clustering) are often used as unsupervised clustering approaches for image processing due to their ease of implementations and outstanding clustering performances. Figure 3 shows the segmentation image for DDSM dataset.

FCMs assist in splitting data into p-dimensional sets X = {x_1_,…, x_i,_….x_n_} (1 ≤ i ≤ n) into clusters (c) by considering their membership degree matrices U = (uti)c * n and suing objective functions J achieving minimal values. utiimplies measures of membership degrees of samples x_i_ related to randonly selected cluster centresV = {v_1_,….v_t_,….v_c_} 1 ≤ t ≤ c which help in identifying clusters. The computations of uti^14,15^ are based on:

3 $${{\text{u}}}_{{\text{ti}}}=\frac{1}{\sum_{{\text{Z}}=1}^{{\text{c}}}{\left({{\text{d}}}_{{\text{ti}}}/{{\text{d}}}_{{\text{zi}}}\right)}^{2/\left({\text{m}}-1\right)}}$$

where *d*_*ti*_ are Euclidean distancesbetween samples*x*_*i*_ andcluster centers*v*_*t*_, *m* signifies power exponents. The Iterations for finding clusters’centreare based on:

4 $$v_{t} = \frac{{\mathop \sum \nolimits_{i = 1}^{n} u_{ti}^{m} d_{ti} }}{{\mathop \sum \nolimits_{i = 1}^{n} u_{ti}^{m} }}$$

where objective functionsare represented by:

5 $${\text{J}} = \sum\limits_{i = 1}^{n} {\sum\limits_{t = c}^{c} {u_{ti}^{m} d_{ti}^{2} } }$$

The main issues in FCM include getting caught in local extreme points or saddle points thus evading optimal solutions. This work handles this issue by modifying the FCM algorithm where weighed Euclidean distances are used in learning of feature-weights. *d*_*ij*_ represent commonly used Euclidean distances, while ${d}_{ij}^{w}$ stands for weighed Euclidean distances, as seen below^16^:

6 $${d}_{ij}^{w}=\sqrt{\sum_{k=1}^{s}{w}_{k}{\left({x}_{jk}-{v}_{tk}\right)}^{2}}$$

Objective functions*J of* Eq. (5) become:

7 $${\text{J}}^{{\text{w}}} \left( {{\text{U}},{\text{ v}}_{{1}} , \ldots .{\text{ v}}_{{\text{c}}} ;{\text{ X}}} \right) = \sum\limits_{i = 1}^{c} {\sum\limits_{j = 1}^{n} {u_{ij}^{m} } } \left( {d_{ij}^{w} } \right)^{2}$$

Then updated *u*_*ij*_, *w*_*k*_ and *v*_*ik*_ are obtained as follows:

8 $${\text{u}}_{{\text{ij }}} = \frac{{n\left( {m\sum\limits_{k = 1}^{s} {w_{k} \left( {x_{jk} - v_{tk} } \right)^{2} } } \right)^{{\frac{1}{m - 1}}} }}{{\sum\limits_{h = 1}^{c} {\sum\limits_{t = 1}^{n} {\left( {m\sum\limits_{k = 1}^{s} {\left( {x_{jk} - v_{tk} } \right)^{2} } } \right)^{{\frac{1}{m - 1}}} } } }}$$

9 $$w_{k} = \frac{{\left( {\mathop \sum \nolimits_{t = 1}^{c} \sum\limits_{j = 1}^{n} {u_{ij}^{m} \left( {x_{jk} - v_{tk} } \right)^{2} } } \right)^{ - 1} }}{{\mathop \sum \nolimits_{h = 1}^{s} \left( {\mathop \sum \nolimits_{t = 1}^{c} \mathop \sum \nolimits_{j = 1}^{n} \left( {x_{jk} - v_{tk} } \right)^{2} } \right)^{ - 1} }}$$

10 $${\text{v}}_{{{\text{ik}}}} = \frac{{\mathop \sum \nolimits_{j = 1}^{n} u_{ij}^{m} x_{jk} }}{{\mathop \sum \nolimits_{j = 1}^{n} u_{ij}^{m} }}$$

The resultant M-FCM algorithm summary is as follows.

- Step 1: Determine max. cluster count c as well as threshold values. Assume m as a suitable constant.
- Step 2: Use FCM to set up memberships and centres.
- Step 3: Using Eq. (9), calculate wk.
- Step 4: Using (7), compute u_(ij)_. Thus, by fresh computed u_(ij)_, update vik according to (10).
- Step 5: Using (7), compute the objective function J_w_.

Stop on convergece or on differences of less than specified thresholds between two consecutive calculated values of objective functions Jw else Go to step 3.

### Feature extraction

The features must be extracted following ROI extraction using CDTM (cross diagonal texture matrix), CDF, and colour histogram, this work extracts texture and form characteristics.

#### CDTM

The characteristics of the texture spectrum (TS) and the diagonal cross texture matrix are combined. The spatial connection between a pixel and the pixels around it at a given angle and distances in textures are represented by GLCM where 8 pixels on the sides of centre pixels are examined using TS texture analyses to produce texture information.

#### CDF

The center distance function will be used as a second contour representation to extract form properties. The distances between boundary points of shapes and centers (x_c_, y_c_) are called center distance functions (Eq. 11).

11 $$R\left(S\right)=\sqrt{{\left({x}_{s}-{x}_{c}\right)}^{2}+{\left({y}_{s}-{y}_{c}\right)}^{2}}$$

#### Color histogram

Due to its intuitiveness in comparison to other qualities and more significant information, simplicity of image extraction, and colour distribution histograms when utilising a set of boxes, colour is the most frequent and commonly utilised property.

Mammographic texture features are associated with breast cancer risk independent of the contribution of breast density. Thus, texture features may provide novel information for risk stratification.

A mammogram image has a black background and shows the breast in variations of gray and white. In general, the denser the tissue, the whiter it appears. This may include normal tissue and glands, as well as areas of benign (noncancerous) breast changes (such as fibroadenomas) and disease (breast cancer).

### Dimensionality reduction using KPCA

For dimensionality reduction, the retrieved features are used as input. A multidimensional input set can be effectively transformed to enhance the algorithm’s overall performance. The ability to condense the whole input space to a significantly lower dimensional subspace is often enabled by the connection between input data and dimensionality reduction. KPCA is employed in the current work to condense the multidimensional F feature space (with D dimension).

KPCA allows for the separability of nonlinear data by making use of kernels. The basic idea behind it is to project the linearly inseparable data onto a higher dimensional space where it becomes linearly separable. KPCA can handle nonlinear relationships between the input features, which can be useful for breast cancer image classification results in better way compare than normal PCA.

### Feature selection using MCSO

After dimensionality reduction, feature selection is done in this work to reduce the time consumption. This work using Mutation CSO for feature selection.

#### CSO (chicken swarm optimization)

CSO is a meta-optimization method with biological roots. The algorithm imitates how a flock of hens might behave and rate one another^17^. A flock of chickens is made up of many groups, each of which has a rooster and a specific number of hens and chicks. For various species of chickens, different movement regulations are applicable^18^. In hens’ social interactions, the pecking order is crucial. The herd's strongest hens will overpower the weaker ones^19^. Near the main cocks, there are more dominating hens, while farther out, there are more submissive hens and roosters.

Traditional CSOs are prone to falling into the local optimisation trap.

To overcome this problem, the mutation operator in CSO was used in this study. This study takes advantage of bit-flip mutations. This mutation operator reverses the bits of the selected genome. (For example, if the gen bit is set to 1 it will be set to 0 and vice versa.).

#### MCSO

The following principles that summarise chicken behaviours form the basis for MCSO:

1. Groups of chickens are separated. There are dominating roosters in groups, followed by hens and younger chicks.
2. Each chicken's fitness score describes its position in the flock hierarchy; The rooster, or each individual, will be the leader, while the individuals with the lowest fitness values will be considered the chicks. Others will be chickens.
3. Within a group, mother–child connections, dominance, and herd hierarchy will not alter. Only a few (G) time steps are required to update these states.
4. The distribution of the N virtual chickens is as follows:

RN, HN, CN, and MNimplysrooster, hen, chick, and mother hen counts represented by their D dimensionallocations.

12 $${x}_{i,j\left(i\in \left[i,\dots \dots ..N\right], j\in \left[1,\dots ..,D\right]\right),}$$

*Rooster Movements* Eqs. (13) and (14) demonstrate that roosters with higher fitness values can forage in more places than those with lower fitness values.

13 $${x}_{i,j}^{t+1}={x}_{i,j}^{t}*\left(1+Randn\left(0,{\sigma }^{2}\right)\right)$$

14 $$\sigma^{2} = \left\{ {\begin{array}{*{20}l} {1,} \hfill & {if\;f_{i} \le f_{k} ,} \hfill \\ {exp\left( {\frac{{f_{k} - f_{i} }}{{\left| {f_{i} } \right| + \in }}} \right)} \hfill & {otherwise\;k \in \left[ {1,N} \right], k \ne i, } \hfill \\ \end{array} } \right.$$

where x_i;j_ imply chosen roostershaving x_i_ as indices Rand n $\left(0,{\sigma }^{2}\right)$ stands for Gaussian distributions with 0 means and ${\sigma }^{2}\mathrm{standard deviations while }\in$ imply small constants values that help in avoiding zero-division-errors, k represents randomly chosen rooster indices selected from groups of roosters, f_i_implies fitness values of corresponding roosters x_i_.

*Hen movement* Chickens follow roosters in search of food. They readily take delicious food that other chickens find when handled by other birds. More dominant hens will win over more submissive hens in competition for food. Examples of mathematical representations of these occurrences are Eqs. (15) and (16).

15 $${x}_{i,j}^{t+1}={x}_{i,j}^{t}+S1*rRand*\left({x}_{{r}_{1,j}}^{t}-{x}_{i,j}^{t}\right)+S2*Rand*\left({x}_{{r}_{2,j}}^{t}-{x}_{i,j}^{t}\right)$$

16 $$S1=exp\left(\left({f}_{i}-{f}_{r1}\right)/abs\left({f}_{i}\right)+\in \right))$$

17 $$S1=exp\left(\left({f}_{{r}_{2}-}{f}_{i}\right)\right)$$

where Rand represents uniform random numbers over [0, 1]. r_1_ $\in$ [1,….., N] represent i^th^ hen’s groupmate roosters’ indices, while r_2_ $\in$ [1,….., N, imply chickens’ (rooster or hen) indices that are randomly selected from swarms.

*Chick movement* Chicks move along with their mothersin search of foods and depicted mathematically as Eq. (18).

18 $$x_{i,j}^{t + 1} = x_{i,j}^{t} + FL*\left( {x_{m,j}^{t} - x_{i,j}^{t} } \right)$$

where ${x}_{m,j}^{t}$ represents positions of ith chicks’ motherswhere m $\in$ [1; N], FL implies parameters representing speedsof chicks following their mothers and to account for differences between chicks, FL values are chosen randomly in the range [0, 2].

The range of each dimension, which is from 0 to 1, is quite broad in feature spaces where features are represented in their own dimensions, necessitating intelligent searches to locate best points. As indicated in Eq. (19), optimal operations of CSOs entail maximisations of classifications on validation sets based on training data while reducing chosen features counts.

19 $${f}_{\theta }=\omega *E+\left(1-\omega \right)\frac{\sum_{i}{\theta }_{i}}{N}$$

where ${f}_{\theta }$ implies fitness functions of given vectors with 0/1 components indicating unselected/chosen features, N represents dataset feature counts, E stands for classifier error rates, and $\omega$ represents constants that control relevance of classifier performances in relation to selected feature counts.

The counts of dataset features correspond to used variable counts in the range [0, 1], and when the values of variables approach 1, the linked attributes become candidates for selections in classifications. The variable, as stated in Eq. (20), is the cutoff that precisely identifies the attributes to be considered in determining each person's fitness.

20 $$f_{i,j} = \left\{ {\begin{array}{*{20}l} 1 \hfill & {if\;X_{i,j} > 0.5} \hfill \\ 0 \hfill & {otherwise,} \hfill \\ \end{array} } \right.$$

where X_ij_ stands for dimensional values of search agents at dimensions j and concurrently update the firefly's locations. As a result, straightforward truncation mechanisms were designed to secure variables' boundaries in the range [0, 1]. Modified values can violate boundary restrictions;

1. Set initial values for RN, HN, CN, MN, G;
2. Set swarm chicken counts randomly
3. X_i_ (i = 1,2,……;N).;
4. Set values for maximumiterationsT_max_;
5. while T < T_max_ do in iterations
6. if T % G = 0 then
7. Classify hens into hierarchical groups based on assessment of their body condition;
8. Divide the flock into groups and determine which groups the mother hens and chicks are connected to;
9. end
10. for each chickens Xi in swarms do
11. if Xi is a roster then
12. update Xi's position using Eq. (14) for each Xi chicken in the flock;
13. end
14. if Xi are hens then
15. Use (16) to modify Xi’s locations;
16. end
17. if Xi are chicks then
18. Use (19) to modify Xi’s locations;
19. end
20. Use Eq. (20) to evaluate the new answer;;
21. If the new solution is superior to the prior one, update it;
22. end
23. end
24. Modify the update solution with flip bit mutation.
25. Evaluate the new solution using Eq. (20)
26. end

### Classification using FCNN

**Figure 4:**
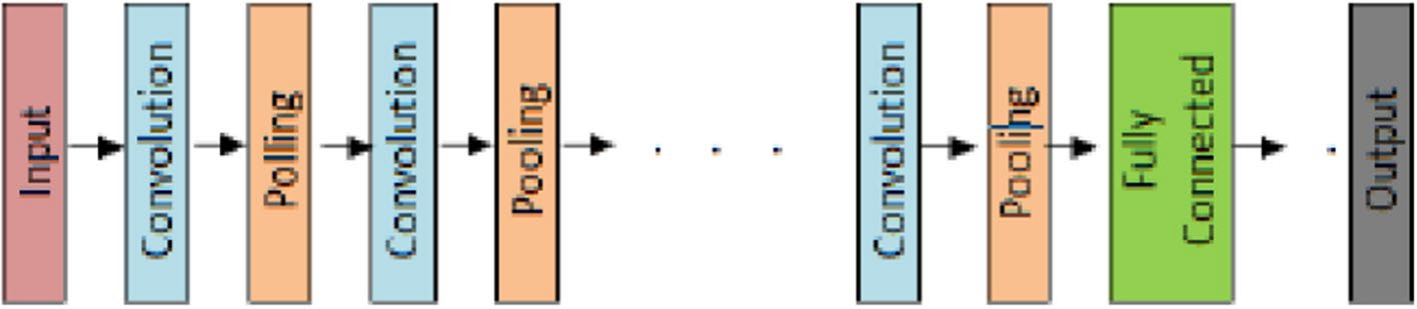
Typical CNN.

Big Data was classified by FCNN following feature choices. In terms of construction, CNN is different from traditional artificial neural networks. The layers of a CNN are chosen to spatially match the input data, unlike a conventional ANN, which flattens the input into a vector. A typical CNN consists of an output layer, one or more convolutional layer blocks anddownsampling layers, and one or more fully connected layers^20,21^. Figure 4 depicts a typical architecture of CNNs.

#### Drawbacks of traditional CNN

Pooling is a technique used by traditional CNN architecture to minimise size, however it can occasionally result in information loss. In order to solve this issue, FCNN was applied in this work.

#### FCNN

**Figure 5:**
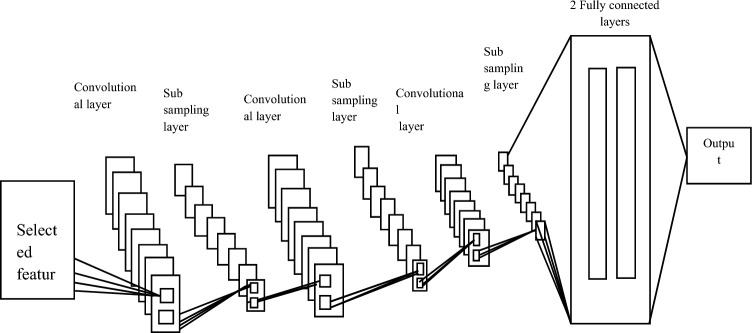
Convolutional neural network architecture.

Three different types of layers are included in FCNN: a convolutional layer, a down sampling layer, and a fully connected layer. The sections that follow provide a brief explanation of each class type. Illustration of a convolutional neural network's architecture in Fig. 5.

#### Convolution layer

In this convolution layer, the input features are merged with the kernel (filter). The n maps of the output feature are generated by convolution of the input feature and kernel. The output features of the convolutional particle and the input are known as feature maps of size i * i, and the kernel of the convolution matrix is known as a filter.

Feature vectors are inputs and outputs of successive convolution layers of CNNs, which contain numerous convolution layers. Each convolution layer is made up of a series of n filters. The number of filters utilized in the convolution process is equal to the depth of the final feature map (n*), and these filters are integrated with the input. It should be noted that each filter map is regarded as a distinct feature at a specific point in the input.

The outputs of *l*-th convolution layers, denoted by ${C}_{i}^{(l)}$, include feature maps which can be calculated using:

21 $$C_{i}^{\left( l \right)} = B_{i}^{\left( l \right)} + \mathop \sum \limits_{j = 1}^{{{a_{i}{\left( {l - 1} \right)}} }} K_{i,j}^{{\left( {l - 1} \right)}} *C_{j}^{\left( l \right)}$$

where, ${B}_{i}^{(l)}$ represents bias matrices and ${K}_{i,j}^{(l-1)}$ stands for convolution filters or kernels of sizes a * *a* that connect *j*-th feature maps in layers (*l* − 1) with *i*-th feature maps in same layers.

Output ${C}_{i}^{(l)}$ layersencompass feature maps. In (22), initial convolutional layers ${C}_{i}^{(l-1)}$ are input spaces or ${C}_{i}^{(0)}={X}_{i}$. Kernels generatefeature maps and subsequently convolution layers’, activationsare applied for their nonlinear transformationoutputs.

22 $${Y}_{i}^{(l)}=Y\left({C}_{i}^{\left(l\right)}\right)$$

where ${Y}_{i}^{(l)}$ is the output of the activation function and ${C}_{i}^{\left(l\right)}$ is the input that it receives.

#### Sub sampling or pooling layer

This layer’s primary goal is to geographically reduce the dimensionality of the feature map that was retrieved from the preceding convolutional layer. The feature map and the mask are subjected to a down sampling procedure. Many other subsampling techniques, including pooling by mean, pooling by sum, and pooling at maximum, have been proposed. Maximum pooling, in which each block’s maximum value corresponds to its associated output characteristic, is the most widely used type of pooling. Be aware that the convolutional layer can tolerate rotation and translation between input images thanks to the down sampling layer.

#### Fully connected layer

**Figure 6:**
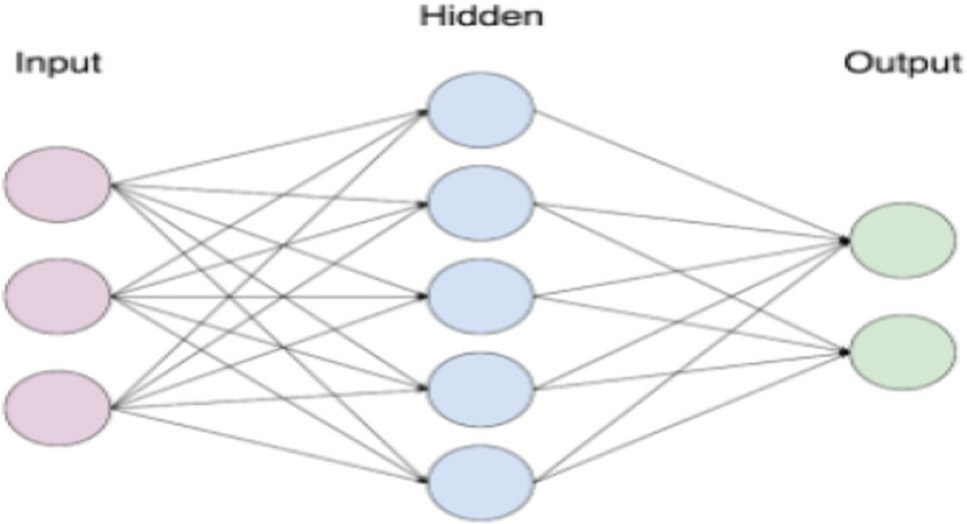
Fully connected layer.

Conventional feedback networks with one or more hidden layers serveas final layers of CNNs. utilising the Softmax activation function as the output layer.

23 $$Y_{i}^{\left( l \right)} = f(z_{i}^{\left( l \right)} )$$

24 $${\text{Where}}\quad z_{i}^{\left( l \right)} = \mathop \sum \limits_{i = 1}^{{{m_{i}{\left( {l - 1} \right)}} }} w_{i,j}^{\left( l \right)} y_{i}^{{\left( {l - 1} \right)}}$$

where ${w}_{i,j}^{(l)}$ are weights that fully connected layers should adjust for producing representations of classes, and f represents nonlinearity transfer functions of fully linked layers’ neurons as seen in Fig. 6 instead of distinct layers as convolutions and pooling layers.

Weights are computed using fuzzy memberships, defined by (${{\text{w}}}_{1}=0.3,{{\text{w}}}_{2}=0.4,{{\text{w}}}_{3}=0.5,{{\text{w}}}_{4}=0.7$) and calculated as:

25 $${o}^{2}={u}_{i}^{\left(j\right)}\left({a}_{i}^{\left(2\right)}\right)$$

where ${u}_{i}^{\left(j\right)}\left(.\right)$ stands for membership functions $u_{i}^{\left( j \right)} \left( . \right):R \to \left[ {0,1} \right]$, i = 1,2,…,M,

j = 1,2,….,N. using Gaussian memberships.

### Fuzzy convolutional neural network parameter optimization using MCSO

In this phase, the network's parameters are simultaneously adjusted, most notably the number of layers, neurons, activation function, optimizer, kernel size, etc. The FCNN's parameters are optimized in this study using Mutation CSO.

## Results and discussion

This section examines the experimental findings using the suggested model. This concept is put into practice using MATLAB. For the MIAS and DDSM datasets, the proposed FCNN model is contrasted with the current Extreme Learning Machine** (**ELM), Kernel Extreme Learning Machine** (**KELM), and MRNN models in terms of accuracy, recall, and error rate. Images and captions for mammograms make up MIAS data. Breast cancer-CNN-Mias/da may be found at (https://www.kaggle.com/code/aditi02). The MIAS digital mammography database contains 350 original images. The DDSM dataset (https://www.kaggle.com/datasets/cheddad/miniddsm2) contains about 1200 images. 70% of the dataset is used for testing, while only 30% is used for training. Table 1 shows the performance comparison results.

**Table 1:** Performance comparison results.

Database	Metrics	Methods			
		ELM	KELM	MRNN	FCNN
DDSM	Accuracy	87.12	92.45	93.33	95.02
	Precision	85.78	91.26	94.56	95.05
	Recall	86.65	90.34	90.13	92.23
	Error rate	14.87	7.55	6.66	4.98
MIAS	Accuracy	88.1	97.85	98.88	98.95
	Precision	90.45	93.09	97.12	98.21
	Recall	91.23	94.54	95.68	96.70
	Error rate	11.9	2.15	1.11	1.05

### Performance metrics

1. Precision

Precisions are percentagesof resultswith relevance and defined as

26 $${\text{Precision}}\;{ = }\frac{{{\text{Truepositive}}}}{{{\text{truepositive}} + {\text{falsepositive}}}}$$

(2) Recall

Recalls are percentages of total relevant results accurately classified by algorithms and defined as

27 $${\text{Recall}} = \frac{{{\text{Truepositive}}}}{{{\text{truepositive}} + {\text{FalseNegative}}}}$$

(3) Accuracy

Accuracies are fractions of right predictions by models and defined as:

28 $${\text{Accuracy}} = \frac{{{\text{Truepositive}} + {\text{TrueNegative}}}}{{{\text{Total}}}}$$

(4) Error rate

Error rates refer to measures of degrees of prediction errors of models with respect to true models and computed as:

29 $${\text{Error rate}} = {1}00 - {\text{Accuracy}}$$

**Figure 7:**
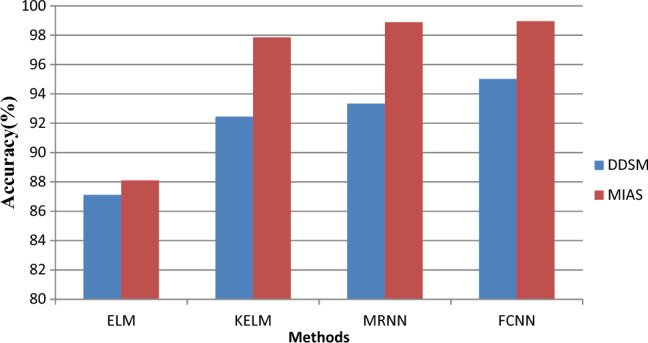
Accuracy results versus classification methods.

The accuracy performance measures of the proposed FCNN are contrasted with those of the current ELM, KELM, and MRNN algorithms in Fig. 7. The chart above x-axis depicts several strategies as well as various methodologies. Precision values are represented by y. The suggested model uses FCNN to categories the data, which introduces fuzzy functions to the process of determining weight values and improves the precision of the findings. The findings indicate that, compared to the present ELM, KELM, and MRNN models, the suggested FCNN model provides a higher accuracy result of 95.02%. For the DDSM dataset, the accuracy is 87.12.45% and 93.33%.

**Figure 8:**
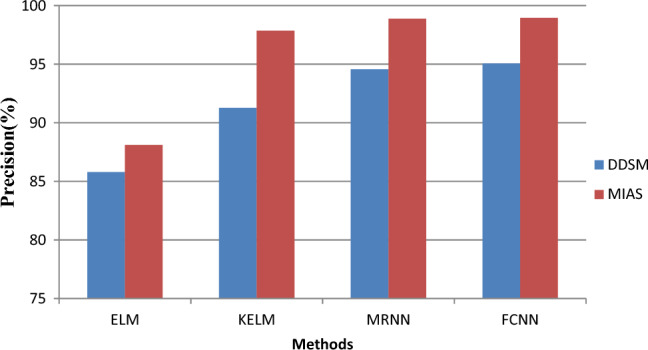
Precision versus classification methods.

The accuracy performance of the proposed FCNN and the existing ELM, KELM and MRNN algorithms are compared in Fig. 8. The above graph shows the different techniques on the x-axis and the accuracy values on the x-axis y. The results show that while the current ELM, KELM, MRNN models only give results with high accuracy, the proposed FCNN model gives the highest accuracy of 95.05%. For the DDSM dataset, precisely 85.78%, 91.26%, and 94.56%.

**Figure 9:**
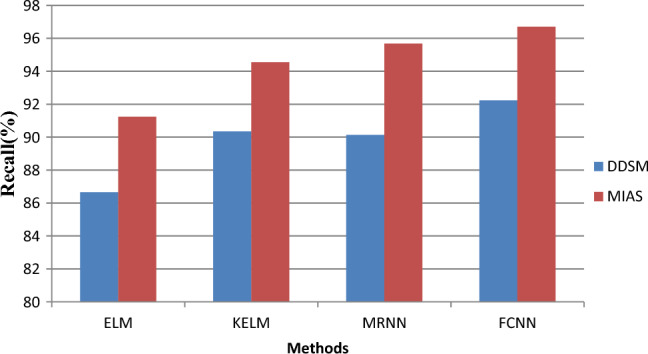
Recall results versus classification methods.

The retrieval performance of the proposed FCNN is compared to the performance of the current techniques ELM, KELM, and MRNN in Fig. 9. The chart above displays the recall values on the y-axis and the various techniques on the x-axis. The suggested FCNN model provides the maximum recovery result (92.23%), according to the findings, while the present ELM, KELM, and MRNN models only produce, respectively, 86.65%, 90.34%, and 90.13% for the DDSM dataset.

**Figure 10:**
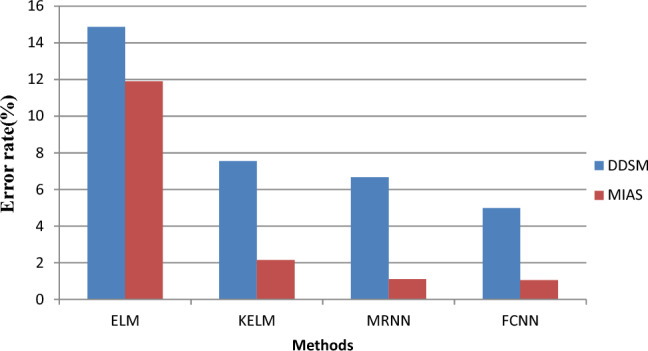
Error rate results versus classification methods.

Figure 10 compares classification errors of FCNN with ELM, KELM, and MRNN methods. The x-axis in the graph above denotes the various methods, and the y-axis denotes a callback of values. The suggested model decreases error rates by constructing an ideal subset of features and chooses significant features based on MCSO utilising mutation operators. The suggested FCNN model has the lowest error rate of 4.98%, according to the results, whereas the current ELM, KELM, and MRNN approaches provide error rates of 14.87%, 7.55 percent, and 6.66% for the DDSM dataset, respectively.

## Conclusion and future work

The most prevalent kind of cancer in women is BC. Increased treatment choices and patient survival are possible with early identification of BC. The gold standard for breast imaging and cancer detection is mammography. With the use of a deep learning model, this effort seeks to develop an automatic BC categorization system. This study uses bilateral filter-based denoising and contrast-stretching-based image enhancement as preprocessing techniques. ROI was derived with MFCM. Block-based CDTM, CDF-based shape descriptors, and colour histograms were used to extract features. For size reduction, KPCA nuclear principal component analysis was utilised. The MCSO is used to choose features. MCSO was used to optimise the FCNN’s parameters after performing the classification. Experimental findings demonstrate that the suggested model outperforms other techniques, providing accuracy of 95.02% for the DDSM dataset and 98.95% for the MIAS dataset. The suggested approach has not yet been used to identify additional illnesses, while this is a potential area for future research.

## Data Availability

The data that support the findings of this study are available on request from the corresponding author.
